# Evaluation of Knowledge and Anxiety Levels in Caregivers of Children With Heart Disease Before and After Basic Life Support Training: A Preliminary Study

**DOI:** 10.7759/cureus.113017

**Published:** 2026-07-20

**Authors:** Anna Dabrowska, Piotr Palczynski, Jaroslaw Meyer-Szary, Karolina Jakitowicz, Mariusz Siemiński, Zuzanna Kusmierz, Joanna Kwiatkowska

**Affiliations:** 1 Student Scientific Circle of Emergency Medicine, Medical University of Gdańsk, Gdańsk, POL; 2 Department of Emergency Medicine, University Clinical Centre, Medical University of Gdańsk, Gdańsk, POL; 3 Department of Paediatric Cardiology and Congenital Heart Defects, University Clinical Centre, Medical University of Gdańsk, Gdańsk, POL; 4 Department of Psychology, Voivodeship Psychiatric Hospital, Gdańsk, POL

**Keywords:** anxiety level, basic life support training, cardiomyopathies, cardiopulmonary arrest, inherited arrhythmia syndromes, knowledge level, parents and caregivers

## Abstract

Introduction

Cardiomyopathies (CMs), as well as primary electrical disorders, i.e., inherited arrhythmia syndromes (IAS), including long-QT syndrome, catecholaminergic polymorphic ventricular tachycardia, and Brugada syndrome, predispose children to malignant ventricular arrhythmias and out-of-hospital cardiac arrest; survival depends on rapid Basic Life Support (BLS) and early automated external defibrillator (AED) use. We evaluated whether a brief, caregiver-focused, arrhythmia-tailored BLS workshop improves knowledge and affects short-term state anxiety.

Materials and methods

The study included 12 caregivers of children with cardiac conditions who participated in BLS training. The study was conducted in three stages: a pre-test (entry survey), intervention (lecture and practical training), and post-test (exit survey). Knowledge was assessed using custom questions based on the ERC 2025 guidelines, and anxiety levels were measured with the State-Trait Anxiety Inventory (STAI). Statistical analysis was performed using Wilcoxon, Mann-Whitney U, and Kruskal-Wallis tests.

Results

The mean knowledge score increased from 5.3 ± 1.0 before training to 7.8 ± 1.0 after training (T = 0.0; p = 0.002). The most significant improvement was observed in practical aspects of resuscitation, such as the compression-to-breath ratio during pediatric CPR. Anxiety levels, measured using STAI-X1, decreased after the training, and the difference was statistically significant (T = 5.5; p = 0.009).

Conclusions

BLS training effectively improves the knowledge of caregivers of children with IAS, which may enhance their competence in emergency situations involving sudden cardiac arrest (SCA). Further research in a larger group is needed to determine the impact of regular and repeated BLS training on long-term knowledge retention, as well as on anxiety levels and quality of life among families of children with CM or IAS.

## Introduction

Cardiomyopathies (CMs) and inherited arrhythmia syndromes (IAS), often referred to as inherited cardiac channelopathies, predispose children and young adults to malignant ventricular arrhythmias and sudden cardiac death (SCD) [1-2]. The major entities include hypertrophic cardiomyopathy (HCM) and arrhythmogenic cardiomyopathy (ACM), while primary electrical diseases include long-QT syndrome (LQTS), catecholaminergic polymorphic ventricular tachycardia (CPVT), and Brugada syndrome (BrS) [1].

In population-based series, up to approximately 30% of SCDs before age 35 are classified as sudden arrhythmic death syndrome (SADS) [1]. Within SADS cohorts, post-mortem genetic testing identifies pathogenic or likely pathogenic variants in approximately 13% overall, rising to approximately 27-39% when expanded cardiac gene testing and systematic evaluation of relatives are incorporated, with channelopathies (LQTS, CPVT, and BrS) comprising a major subset [2-4].

Such events frequently occur in the community, during exertion or emotional stress in LQTS/CPVT and during fever or rest in BrS [5-7]. These trigger patterns underscore the need for tailored anticipatory guidance and emergency action plans for families, as timely recognition of unresponsiveness and rapid activation of emergency services are critical.

Because arrhythmias in IAS are often shockable, outcomes hinge on the pediatric “chain of survival”: early recognition and EMS activation, high-quality basic life support (BLS), and prompt defibrillation using an automated external defibrillator (AED) [8]. Observational data and a systematic review indicate that lay-rescuer AED application is associated with higher survival and better neurological outcomes in pediatric out-of-hospital cardiac arrest, and survivors are more likely to have received bystander defibrillation [9-10]. Accordingly, brief, targeted BLS/AED training for parents and caregivers of children at risk of SCD is a plausible, scalable strategy to improve readiness for time-critical events.

Chronically ill patients at risk of SCD, as well as their families, may experience ongoing chronic anxiety. We hypothesize that BLS training sessions can significantly affect caregivers' sense of competence and reduce anxiety levels among families of chronically ill children. The study aimed to assess the impact of BLS training on the knowledge and anxiety levels of caregivers of children with CMs and IAS.

## Materials and methods

Study design

**Figure 1:**
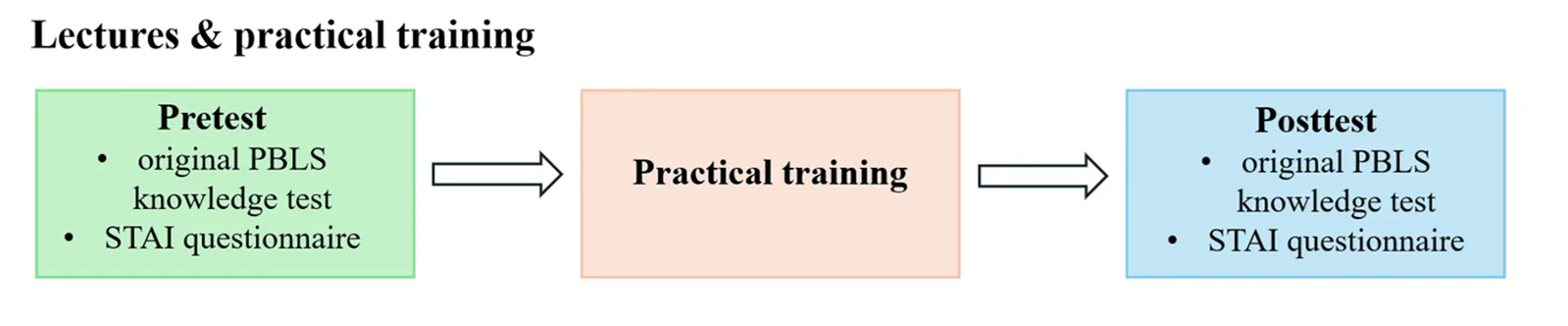
Stages of the study. Stage 1: Pretest questionnaire assessing PBLS knowledge and anxiety level. Stage 2: Educational intervention including theoretical instruction and practical CPR/AED training. Stage 3: Immediate posttest questionnaire evaluating knowledge and anxiety level after the training. PBLS: Pediatric basic life support; CPR: Cardiopulmonary resuscitation; AED: Automated external defibrillator; STAI: State-Trait Anxiety Inventory.

The study was divided into three stages (Figure 1). The first stage consisted of completing an entry survey (pretest). During the second stage, the intervention, participants underwent training that included a lecture and practical exercises. Practical classes were conducted simultaneously in subgroups and included exercises in basic rescue procedures using CPR mannequins and an AED. The training was conducted by physicians from the Department and Clinic of Emergency Medicine, as well as the Department of Paediatric Cardiology and Congenital Heart Defects of the Medical University of Gdansk. The third stage involved completing the exit survey (posttest) immediately after the training to compare the participants' knowledge levels.

Both surveys were identical and consisted of two parts: (A) anxiety assessment and (B) BLS knowledge test. The anxiety assessment was performed using the State-Trait Anxiety Inventory (STAI; Spielberger's State-Trait Anxiety Inventory) questionnaire in the Polish adaptation [11]. The STAI questionnaire consisted of two parts, each with 20 statements, assessing anxiety as a state (STAI X-1) and anxiety as a personality trait (STAI X-2). The statements were rated on a four-point scale, with a maximum score of 80 points in each part. According to the STAI, anxiety can be divided into three categories: low (20-39 points), moderate (40-59 points), and high (60-80 points). The test consisted of 10 original questions assessing knowledge of pediatric BLS, arranged based on the ERC Guidelines 2025. The survey was available both before and after the training, in print and online.

Participant recruitment process

Twelve parents and caregivers of children with cardiac conditions, including CMs or channelopathies such as LQTS, participated in the training. Patients with a diagnosis of IAS or CM were selected from the genetic database of the Department of Paediatric Cardiology and Congenital Heart Defects. The following factors were considered: family history, clinical presentation, genetically confirmed diagnosis, and a history of cardiac arrest or increased risk of sudden cardiac death. Of the several dozen caregivers invited to participate in the study, 12 agreed.

Ethical considerations

The independent Bioethics Committee for Research at the Medical University of Gdansk (KB/232/2026) gave a positive opinion on the project and the research tool. Participation in the study was voluntary, and at any stage of the project, participants had the opportunity to withdraw from the study.

Statistical considerations

Statistica v.13.3 (TIBCO Software Inc., California, USA) was used for statistical analysis. The Wilcoxon test, Mann-Whitney U test, and Kruskal-Wallis test were applied to analyze differences between variables, with a significance level of p < 0.05.

## Results

**Figure 2:**
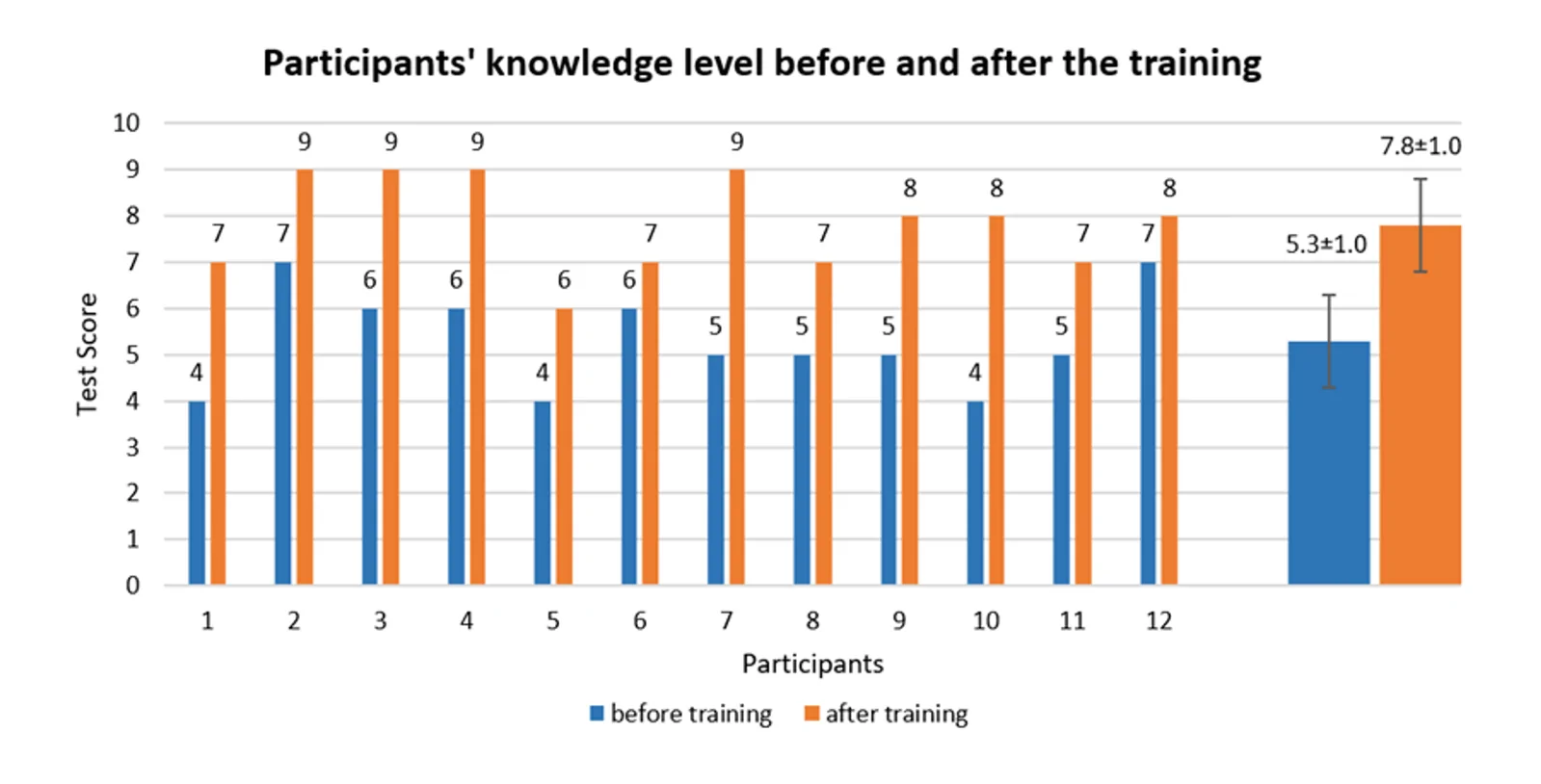
Increase in participants’ knowledge scores after the training.

The 12 people who completed the survey both before and after the training were included in the comparative analysis. Most respondents were women (N = 9; 75%) and were in the age group of 30-50 years (N = 7; 58%). The average pretest score was 5.3 ± 1.0 points, ranging from four to seven points (median = 5). In turn, the average posttest knowledge score was 7.8 ± 1.0 points. The lowest knowledge score was six points, and the highest was nine points (median = 8) (Figure 2). The Wilcoxon test indicated a statistically significant difference in the results (T = 0.0; p = 0.002). There were no significant differences in knowledge scores between women and men before (5.3 ± 1.2 vs. 5.3 ± 0.5; U = 13.0; p = 1.000) and after the training (7.8 ± 1.0 vs. 8.0 ± 0.8; U = 12.0; p = 0.853). Moreover, no significant differences were observed between age groups and knowledge scores before (H = 1.51; p = 0.47) or after training (H = 0.91; p = 0.635).

Before the training, the study participants had insufficient knowledge of cardiopulmonary resuscitation in children. The most difficult question concerned the first action to take if a child loses consciousness and stops breathing while eating. Only one respondent (8%) answered correctly that CPR should be started according to the pediatric BLS algorithm. Seven participants (59%) indicated upper abdominal compressions to “push” the foreign body out of the respiratory tract, three (25%) chose to perform five back blows, and one (8%) chose to open the child's mouth and remove the foreign body with a finger. Moreover, around one-third of the respondents (N = 4; 33%) incorrectly indicated the compression-to-breath ratio during pediatric CPR. In contrast, participants most frequently correctly indicated that movement and coughing are symptoms of return of spontaneous circulation (ROSC; N = 11; 92%) and who can use the AED (N = 11; 92%).

After the training, improvement was observed in seven questions. All respondents knew what to do if the chest did not rise despite rescue breaths (N = 12; 100% vs. N = 8; 67% before the training) and who can use the AED (N = 12; 100% vs. N = 11; 92% before the training). Another easy question concerned situations in which the AED should not be used. All respondents selected the correct answer (N = 12; 100% vs. N = 5; 42% before the training). Ten respondents (83% vs. N = 4; 33% before the training) knew how long to check a child's breathing, and almost all correctly indicated when to stop chest compressions (N = 10; 83% vs. N = 9; 75% before the training). After the training, the respondents were able to correctly indicate the compression-to-breath ratio during pediatric CPR (N = 12; 100% vs. N = 4; 33% before the training). The respondents provided the correct answer with the same frequency as before the training for two questions: symptoms of ROSC (N = 11; 92%) and who can perform CPR on a child (N = 9; 75%). The most challenging question concerned the initial step in CPR in children with SCA. None of the participants answered correctly (N = 0; 0% vs. N = 2; 17% before the training). Table 1 shows the questions and the number of correct answers.

**Table 1:** Questions with correct answers and the number and percentage of correct answers before and after the training. ¹ Because only one participant changed their answer after the training, the Wilcoxon test could not be reliably calculated; therefore, the T and p values are not reported. AED: Automated external defibrillator; CPR: Cardiopulmonary resuscitation; BLS: Basic life support; ROSC: Return of spontaneous circulation; p: p-value; T: Wilcoxon test statistic.

No.	Question	Correct answer	Before training	After training	p	T
1	Who can use an AED (automated external defibrillator)?	Anyone, without restriction	11 (92%)	12 (100%)	-¹	-¹
2	When can we stop chest compressions?	All of the above: when the emergency medical team arrives at the scene and takes over rescue operations; when the rescuer becomes exhausted; or when signs of life return	9 (75%)	10 (83%)	-¹	-¹
3	How do we start CPR in a child with sudden cardiac arrest?	Use an AED	2 (17%)	0 (0%)	0.18	0
4	Who can perform CPR on a child?	Anyone who witnesses a cardiac arrest	9 (75%)	9 (75%)	1	1.5
5	How long should we check a child's breathing if cardiac arrest is suspected?	10 seconds	4 (33%)	10 (83%)	0.028	0
6	Which compression-to-breath ratio should be used when performing CPR on a child?	15 compressions followed by two rescue breaths	4 (33%)	12 (100%)	0.012	0
7	While eating, the child suddenly became pale, lost consciousness, stopped breathing, and turned blue. What should we do first?	Start CPR according to the pediatric BLS algorithm	1 (8%)	6 (50%)	0.091	4
8	When should we not use an AED?	None of the above: in a person with a pacemaker, in a pregnant woman, or in a person after drowning	5 (42%)	12 (100%)	0.018	0
9	What should we do if we do not see the chest rising despite performing rescue breaths, assuming we have opened the airway properly and there is no visible obstruction in the airway?	Move on to chest compressions	8 (67%)	12 (100%)	0.068	0
10	What are the symptoms of ROSC?	Movement and coughing	11 (92%)	11 (92%)	-¹	-¹

The STAI-X1 analysis (anxiety as a state) showed that anxiety averaged 43.4 ± 8.3 points before the training, indicating moderate anxiety, and 39.0 ± 7.4 points after the training, indicating low anxiety. A low anxiety level was found in seven respondents (58%) after the training. The Wilcoxon test showed that the difference in results was statistically significant (T = 5.5; p = 0.009). In the STAI-X2 questionnaire (trait anxiety), the average score was 47.7 ± 10.0 points before the training and 46.0 ± 9.4 points after the training. In both cases, this indicated moderate anxiety (Table 2). Low anxiety levels were observed in five participants (42%) after the training. No statistically significant difference was observed between the scores (T = 23.5; p = 0.224). The results obtained using the STAI-X1 questionnaire did not show a significant association between age and anxiety level, either before (H = 3.99; p = 0.136) or after the training (H = 3.69; p = 0.158). Similarly, analysis of the STAI-X2 questionnaire did not show a significant association between age and trait anxiety level before (H = 2.68; p = 0.262) or after the training (H = 5.57; p = 0.062). Gender did not influence anxiety levels before the training (STAI-X1: U = 7.0; p = 0.267; STAI-X2: U = 8.0; p = 0.355) or after the training (STAI-X1: U = 10.0; p = 0.579; STAI-X2: U = 9.0; p = 0.46).

**Table 2:** Results for the individual parts of the STAI questionnaire. STAI: State-Trait Anxiety Inventory; STAI-X1: State Anxiety Inventory; STAI-X2: Trait Anxiety Inventory; p: p-value; T: Wilcoxon test statistic.

STAI component	Training	Minimum	Maximum	Median	Mean	SD	p	T
State anxiety (STAI-X1)	Before	33	57	41	43.4	8.3	0.009	5.5
	After	28	56	38	39	7.4		
Trait anxiety (STAI-X2)	Before	36	64	45.5	47.7	10	0.224	23.5
	After	33	62	48	46	9.4		

## Discussion

The BLS training conducted in this study, based on the ILCOR consensus [12], showed a significant increase in knowledge among parents/caregivers of children with IAS. The initial knowledge of parents in our study was, on average, 5.3 points, which increased to 7.8 points after training. Similar results have been noted in the general population, with training typically increasing participants' knowledge by 30-50% [13]. A systematic review by Cartledge S et al. also confirms the high effectiveness of BLS education aimed at families of high-risk cardiac patients [14].

The baseline knowledge of parents in our study was relatively low, aligning with findings from other research. Global analyses indicate a generally low level of knowledge regarding BLS, particularly among families of cardiac patients [15-16]. Notably, families of children with IAS often have limited knowledge of first aid, despite the high risk of SCA in their children [17-18].

The improvement in knowledge observed after our training aligns with findings from other studies on specific populations. Some studies have demonstrated significant knowledge improvements among parents of infants with heart defects [19-20]. Knight LJ et al. reported that all parents of high-risk children declared an increase in CPR competencies following training [21]. Similar outcomes were observed in studies by Han KS et al. and Kim HS et al., who noted significant increases in knowledge and competencies among families of cardiovascular disease patients after targeted CPR training [22-23].

In our survey, the greatest progress was observed in questions concerning practical aspects of resuscitation, such as the time required to check for breathing or recognizing signs of ROSC. However, one key question, regarding the initial step in CPR for children with SCA, remained challenging both before and after the training. This result suggests the need to place greater emphasis on this particular aspect in future training sessions.

Despite the improvement in knowledge, anxiety levels in our study did not decrease uniformly across all STAI domains. State anxiety decreased after the training, whereas trait anxiety remained moderate both before and after the training. These findings suggest that anxiety among caregivers of chronically ill children, especially those with CM and IAS, may be more related to chronic stress than directly to a lack of CPR knowledge [24-26]. Abdel-Salam A et al., however, noted that educational programs could improve quality of life and self-efficacy among mothers of children with CM and IAS, indirectly influencing anxiety reduction [27].

Although the training recipients came from families in which SCA had occurred, not only in children but also in adults, or in which the risk of SCA was significantly higher than in the general population, the results suggest the need to improve BLS implementation skills in this group. During the sessions, the researchers recognized the unique nature of the situation, as most participants were either survivors of SCA, witnesses of SCA in a close family member, or had an implanted cardioverter-defibrillator as a preventive measure against SCA. Introducing cyclic training sessions and repeating them at regular intervals could contribute to better retention of acquired knowledge and enhanced competencies among participants. Allocating specific resources within the healthcare system to address the needs of these particular patient groups and their families should be a subject of further analysis.

The study has several limitations. One of the primary limitations was the small sample size, with 12 participants included in the final analysis, making the study underpowered to show changes of lesser effect size. Second, the small sample size, in the setting of heterogeneity in the study population, including variations in age, education level, and prior first aid experience, may have led to sampling or selection bias that influenced the outcomes both before and after the training. Third, the single-centre design may limit the generalizability of the results. Moreover, many caregivers who were invited to participate in the study did not join because they were afraid or unwilling to travel to the research centre. Before future studies, we plan to provide better psychological preparation and information to prospective participants in order to reduce anxiety and encourage participation.

## Conclusions

In this population, early defibrillation is a major determinant of survival, yet outcomes still depend heavily on the presence and actions of lay bystanders. The skills acquired during BLS training are particularly important because they increase the chances of survival for children experiencing cardiopulmonary arrest.

Our study shows that tailored, caregiver-focused BLS training significantly improves the knowledge of parents of children with CM and IAS, consistent with the findings of other studies.

Anxiety among parents of children with CM and IAS is a prevalent phenomenon that has a negative impact on the quality of life of the entire family. The study showed that BLS training had a positive effect on caregivers' knowledge. It remains to be further evaluated whether regular repetition of BLS courses may have an effect on long-term knowledge retention, as well as on the anxiety levels and quality of life of patients and their caregivers. In the long term, this could lead to lower stress levels and improved quality of life for the entire family. Further research in a larger group is needed to determine more precisely the impact of BLS training and factors such as age or gender on anxiety levels.

## Appendices

Appendix 1

Questionnaire

1.  Who can use an automated external defibrillator (AED)?

a) Qualified medical personnel

b) Anyone after appropriate training, such as during a first aid course

c) Anyone without restriction

d) Only a physician

2. When can we stop chest compressions?

a) When the emergency medical team arrives at the scene and takes over rescue operations

b) When the rescuer becomes exhausted

c) When we notice the return of signs of life

d) All of the above

3. How do we start CPR in a child with sudden cardiac arrest?

a) Rescue breaths

b) Chest compressions

c) Use of an AED

d) By calling for help, because CPR in a child may be performed only by qualified personnel

4. Who can perform CPR on a child?

a) Only a parent

b) Only medical personnel

c) Anyone who witnesses a cardiac arrest

d) Anyone who has completed a first aid course

5. How long should we check a child's breathing if cardiac arrest is suspected?

a) 10 seconds

b) 15 seconds

c) 30 seconds

d) 60 seconds

6. Which compression-to-breath ratio should be used when performing CPR on a child?

a) 15 compressions followed by 2 rescue breaths

b) 30 compressions followed by 2 rescue breaths

c) 15 compressions followed by 1 rescue breath

d) 50 compressions followed by 2 rescue breaths

7. While eating, the child suddenly became pale, lost consciousness, stopped breathing, and turned blue. What should we do first?

a) The child has probably choked; I will open the child's mouth and remove the foreign body with a finger

b) I will perform upper abdominal compressions to “push” the foreign body out of the respiratory tract

c) I will perform 5 back blows between the shoulder blades

d) I will start CPR according to the paediatric BLS algorithm

8. When should we not use an AED?

a) In a person with a pacemaker

b) In a pregnant woman

c) In a person after drowning

d) None of the above

9. What should we do if we do not see the chest rising despite performing rescue breaths, assuming we have opened the airway properly and there is no visible obstruction in the airway?

a) I will move on to chest compressions

b) I will try again, delivering a larger volume of air

c) I will try three more times. If there is still no effect, I will move on to chest compressions

d) I will call for help as quickly as possible and stop chest compressions because compressions will be ineffective without rescue breaths

10. What are the symptoms of ROSC?

a) Movement

b) Coughing

c) Ineffective gasping

d) A and B are correct
